# Impact of Multidisciplinary Cleft Team Care on Oral Health Quality of Life in Children With Unilateral Cleft Lip and Palate: A Focus on Early Intervention vs. Sporadic Treatment

**DOI:** 10.1155/ijod/1642111

**Published:** 2025-03-19

**Authors:** Shabnam Ajami, Mahtab Ebrahimi Nezhad, Faezeh Bahraini, Nasser Nadjmi, Maryam Zeraatkar

**Affiliations:** ^1^Orthodontic Research Center, Shiraz University of Medical Sciences, Shiraz, Iran; ^2^Department of Cranio-Maxillofacial Surgery, Faculty of Medicine and Health Science, University Hospital, University of Antwerp, Antwerp, Belgium; ^3^Department of Dental Public Health, School of Dentistry, Shiraz University of Medical Sciences, Shiraz, Iran

**Keywords:** cleft lip, cleft palate, preschool children, quality of life, surgery

## Abstract

**Objective:** This is a study evaluating oral health-related quality of life (OHRQoL) using the Farsi version of Early Childhood Oral Health Impact Scale (F-ECOHIS) in children with unilateral cleft lip and palate (UCLP) who were treated at a multi-disciplinary cleft center, adopted from another surgeon, and the ones did not have clefts and were treated at that dental clinic.

**Design:** Cross-sectional study.

**Setting:** The study was conducted at the Lip and Palate Cleft Clinic (Orthodontic Research Center, Shiraz University of Medical Sciences).

**Patients:** The participants were enrolled from the Lip and Palate Cleft Clinic and the Department of Pediatric Dentistry.

**Main Outcome Measures:** The OHRQoL of preschool children and their caregivers.

**Results:** The total score of (ECOHIS) in all subscales of both domains of child impact and family impact was statistically lower for the study group than the other groups. Two by two comparisons showed significant differences between the team-managed and non-team groups (*p* < 0.001). In any domain subscale, there were no gender differences among the three groups (*p* > 0.05).

**Conclusions:** The team-managed group obtained better scores in all subscales in comparison to the control and the non-team patient groups; however, since randomization and a controlled surgical method were not possible, the improvements in quality-of-life scores cannot be directly related to the surgical method.

## 1. Introduction

Cleft lip and palate (CLP), the most common congenital craniofacial condition, requires multidisciplinary treatment and involves numerous interventions and regular follow-ups throughout the patient's youth [1]. A variety of health complications, including dento-skeletal malocclusion, facial deformities, speech impairment, feeding problems, and hearing difficulties, might accompany CLP. The compromised esthetic and functional well-being associated with CLP often requires frequent clinical visits and, in some cases, surgical interventions, which can impose additional stress on affected children and their parents [2–4]. While most primary surgical repairs are completed within the first 2 years of life, ongoing evaluations and rehabilitation interventions typically continue throughout the school-age years. This prolonged and complex cleft management process has led to CLP being regarded as a chronic condition, significantly affecting the quality of life of individuals and their families [5, 6]. The outcomes of medical treatments are often evaluated in terms of improvements in the quality of life of patients with a history of chronic diseases [7]. Previous studies have shown that CLP can significantly impair quality of life [8–10]. Various techniques have been introduced for repairing different CLP, but the ideal surgical technique is still unknown. A systematic review of the studies comparing the palatal closure techniques concluded that the Sommerland and Furlow palatoplasty approaches led to the best results for middle ear function and speech [11]. According to a European cohort study, the nasolabial appearance of the affected ones had improved steadily over the past five decades following the improvements in surgical techniques for treating CLP [12]. The effect of surgical treatment on improving QoL of children and their caregivers has been reported when comparing before and after surgical procedures [13].

An additional method to assess the effect of oral health on the quality of life is the oral health quality of life (OHRQoL) questionnaires [14]. Studies have developed different questionnaires to assess OHRQoL for individuals >6 years of age, but an age-appropriate questionnaire is paramount for younger children [15]. The Early Childhood Oral Health Impact Scale (ECOHIS) has been used to determine the burden of oral diseases among preschoolers. Many studies have used ECOHIS to demonstrate the effects of early childhood caries, tooth trauma, and malocclusion [16–18]. A few studies have revealed that the OHRQoL of children and their guardians could be adversely affected by the presence of CLP [7, 19, 20]. The CLP, as the most common condition in the facial region, can have a tremendous impact on all aspects of the lives of the affected individuals and their families. Sischo et al. stated that preschool children with CLP had been neglected in quality-of-life evaluations. These years are some of the most critical ones in psychological development. While preschool children with CLP strive for autonomy and start to experience more social interactions, the differences in appearance and some functional limitations may lead to vulnerability [21, 22].

Many other studies also evaluated the OHRQoL of older children with CLP compared to those not affected, using different types of questionnaires that can be applied for that specific age range, demonstrating the impact of cleft on the OHRQoL of the affected children [23–25]. A team approach for management combined with more contemporary surgical methods for repairing CLP was considered to improve the esthetic and functional requirements when physical and psychological effects of oral health were evaluated [7].

Due to the high incidence of CLP in the southeast of Iran and being the referral center for the affected individuals, the Shiraz University of Medical Sciences developed a CLP clinic at the Orthodontic Research Center in 2011. This clinic is responsible for the management of the newborns with CLP. Therefore, guidelines for multidisciplinary care were developed to achieve better outcomes. The techniques used in the early years for lip and palatal repair were Millard's rotation advancement and V-Y push-back palatoplasty, respectively. However, in recent years, the surgical techniques have been shifted to different ones having modified Millard's rotation for lip repair and modified Furlow palatoplasty performed by their pioneers for the affected ones [26].

The authors aim to highlight team management as the central factor while acknowledging the role of improved surgical techniques. The focus is on demonstrating that professional, multidisciplinary team management, combined with advancements in surgical methods, leads to improved OHRQoL in children with CLP.

However, the hypothesis specifically emphasizes the *impact of team management*, proposing that children under the supervision of a professional team are likely to experience better OHRQoL outcomes. While surgical advancements are considered, they are presented as complementary to the coordinated efforts of the multidisciplinary team.

## 2. Materials and Methods

The Ethics Committee at Shiraz University of Medical Sciences approved this study under the ethical code IR.SUMS.REC.1395.S662 and IR.SUMS.REC.1401.108. Caregivers of the patients who attended their routine follow-ups at the Lip and Palate Cleft Clinic (Orthodontic Research Center, Shiraz University of Medical Sciences) from 2013 to 2019 were invited to participate in this study by the cleft team members.

### 2.1. The Characteristics of the Participants Included

**Figure 1:**
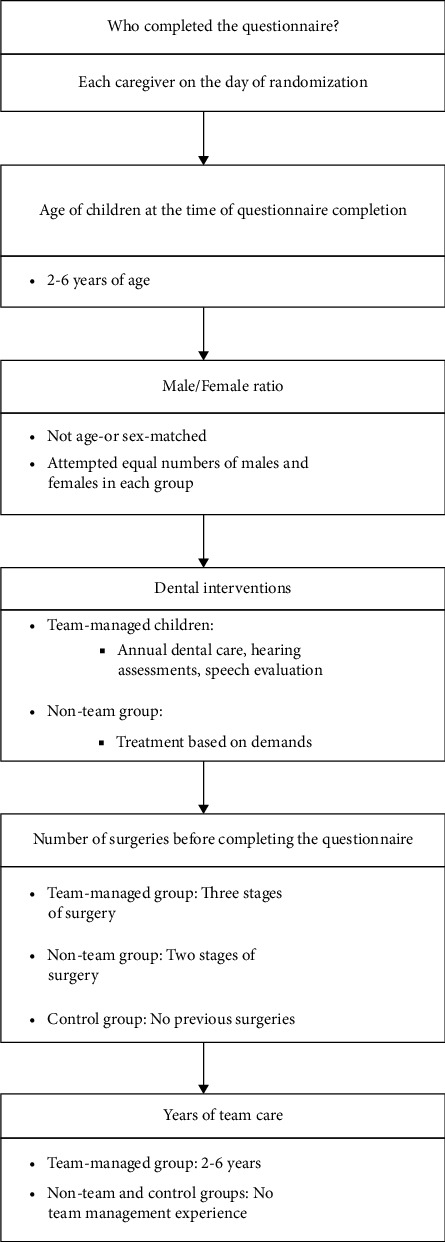
Descriptive information about participants.

The present study included three groups: with 2–6-year-old children. In all the groups, there were no other syndromes, history of severe trauma to the head and neck area, or systemic diseases [27]. The selected groups were as follows:

1. The team-managed group: 30 children with a history of non-syndromic unilateral complete cleft lip and palate (UCLP) were collected using a convenience sampling technique from those treated by the multidisciplinary team management from birth and prior to any surgery. Team members held multiple sessions to assess each affected child and decide on the required procedures. They all underwent modified Millard's rotation advancement for lip repair and two stages of modified Furlow technique for their palatal closure after a short survey by all the experts in the field. Children under team management received regular follow-ups from birth. Parents received psychological counseling once after birth when they referred to us. Their hearing ability was assessed every 3 months using audiometry, tympanometry, and auditory brainstem response (ABR) testing—specifically for children under 3 years of age and those older than 3 years, respectively. Early speech intervention techniques were introduced to caregivers following the primary surgeries. Again, the speech was evaluated 1 month after surgery, and in case of any problem, they had sessions of speech therapy. A nurse specialist in CLP taught feeding to caregivers at birth, and they received oral hygiene instruction at 6 months of age. Oral hygiene was checked annually and the children underwent the preventive treatments, and in case of any problem, they were referred to a pedodontist. Caregivers kept in touch with each other via social media and benefited from each other experiences.
2. The non-team patient group: Thirty-one 2–6-year-old children with a history of non-syndromic unilateral complete CLP were enrolled by our team after their surgical repair procedures before alveolar bone grafting. Until referral, these children had not undergone any routine speech or hearing ability follow-ups, oral hygiene instructions, or feeding training. They only had sporadic treatments based on the symptoms, or urgent referrals by the family physicians. They were treated with the conventional Millard's rotation advancement technique for lip repair and a single-stage palatal closure with V-Y push-back palatoplasty by an individual provider.
3. Control group: Thirty children without CLP or any other anomalies with a similar age range and sex, who attended the Department of Pediatric Dentistry for routine dental follow-ups, were considered as the control group. This group was evaluated using F-ECOHIS questionnaire before receiving any dental care. Some detailed information about the participants is shown in Figure 1.

### 2.2. Data Collection

In this study, the OHRQoL of all the participants was assessed using the Persian version of the ECOHIS (F-ECOHIS), which reliability and validity were determined by Jabarifar et al. [22]. The parents and caregivers answered this questionnaire to determine their perception on the OHRQoL of their 2–6-year-old children. After signing the consent forms and being assured about the privacy and confidentiality of the gathered data, the caregivers individually answered the questionnaire in a private room in the Cleft Lip and Palate Clinic, Orthodontic Research Center, Shiraz University of Medical Sciences. Non-team-managed families completed the F-ECOHIS at their first team visit. The authors confirm that all the methods were performed in accordance with the relevant guidelines and regulations and in line with the Declaration of Helsinki.

The questionnaire included 13 questions in two main domains, of which nine evaluated the impact on the child, and four assessed the impact on the family. The section related to the impact on children was divided into four subscales:

1. 1Pain-related symptoms
2. Limitations related to functions such as speech, eating, and drinking and also missing daycare.
3. Psychological impacts, including sleeping disorders and getting annoyed
4. An aspect of self-image that assesses smiling and social avoidance.

The impact on the family was divided into two subscales:

1. Parental distress, including feeling guilty and annoyed
2. Family functions related to missing work and financial problems.

The chosen response for each subscale was scored on a 5-point Likert scale from 0 to 5, starting with “never,” followed accordingly by “hardly ever,” “occasionally, often, very often,” and “do not know.” The subscale scores were determined by adding up the responses for each item. The response for impact on the child and family domains had scores ranging from 0 to 36 and 0 to 16, respectively. The total OHRQoL score was calculated by summing up all the subscales of both domains. The higher the scores were, the more negative impact on OHRQoL could be reported, and vice versa.

### 2.3. Data Analysis

Data were reported using the median and interquartile range (Q_1_–Q_3_). The Kolmogorov—Smirnov test was used to assess the normality of the data. Since the data were nonparametric, the Kruskal–Wallis test was applied to compare the median (Q1Q3) of the F-ECOHIS scores among the groups. Kruskal–Wallis test was used for multiple comparisons. The reliability coefficient was assessed by Cronbach's alpha. The data were analyzed using SPSS 22 (SPSS Inc., Chicago, IL., USA). The significance level was set at *p* < 0.05.

## 3. Results

The reliability of the study was assessed via Cronbach's alpha, which was 0.87. Ninety-one questionnaires were collected and studied for the final analysis. Thirty participants were caregivers of patients with non-syndromic UCLP managed by the cleft team for surgical repairs (53% girls and 46% boys), 31 were non-syndromic UCLP children referred to the team after conventional primary surgeries (43% girls and 57% boys), and 30 children without CLP and without any other craniofacial anomalies were assigned as the control group (50% girls and 50% boys). There were no significant differences in any domain subscales between boys and girls among all the groups (*p* > 0.05), so the data were pooled. See Table S4 in Supporting Information.

### 3.1. Comparison of Total F-ECOHIS Scores

According to the perception of the caregivers, the total score of ECOHIS for the children in the non-team group was significantly higher compared to the team-managed and the control group (*p* < 0.001) (Table 1). There was no significant difference in total ECOHIS between the control group and the team-managed group (*p* > 0.05) (Table 2).

### 3.2. Comparison of the Subscale Scores

The scores of all subscales for the children in the non-team group were significantly higher compared to the team-managed and the control group (*p* < 0.001) (Tables 3 and 2). Compared to the control group, the team-managed group showed no significant differences in any child impact domains except the subscale of child psychological and limitation impact, in which the control group had higher scores (*p* < 0.05) (Table 3). The median score of the control group was higher than the team-managed group.

## 4. Discussion

Although treatments vary for different chronic conditions, clinical examination, teamwork management, and hospitalizations are all stress-inducing factors for the caregivers and patients. CLP is a chronic condition requiring many therapy sessions and multiple surgical treatments in the first few years after birth. While team members strive to improve facial esthetic, speech, and hearing functionalities, assessing patient-oriented outcomes is highly valuable. OHRQoL questionnaires are one of the tools applicable to measure the outcomes in children with CLP.

In the present study, we found significantly better scores in OHRQoL in children with UCLP in the team-managed group.

Multidisciplinary Care Reduces Burden on Caregivers and Improves Outcomes. Our study showed that children managed by a multidisciplinary team had better OHRQoL scores compared to those receiving sporadic or non-team care. This aligns with previous studies, such as Webb et al., which emphasized that structured team-based approaches result in better functional outcomes with fewer future interventions. Team-managed children and their caregivers undergo regular evaluations by a cleft team comprising surgeons, orthodontists, pediatricians, and other specialists, ensuring comprehensive care. The improved OHRQoL scores in our study suggest that regular follow-ups, preventive measures, and coordinated care substantially reduce the psychological and logistical burdens on caregivers [29].

Significant differences were noted between the groups in both family and child impact domains. The lowest scores in the family impact domain were observed in the team-managed group, likely due to the structured support provided by the team. A reduction in the burden of dental treatments and the provision of preventive care and hygiene instructions to caregivers contributed to these results. This finding is supported by earlier research, such as a study using the ECOHIS, which found improvements in family and child domains following primary surgical treatments [28]. On the other hand, in contrast to our study, Defabianis et al. evaluated the OHRQoL using the Child Oral Health Impact Profile (COHIP) and showed no differences in scores for QoL according to the surgical protocols [30].

Within the child impact domain, team-managed children had better scores in all subscales compared to non-team and control groups, with significant differences observed in the child psychology subscale. However, despite better overall scores, lower scores in the child psychology subscale in the team-managed group suggest that surgical techniques or psychosocial support might still need refinement. While multidisciplinary management undoubtedly enhances quality of life, ongoing psychological support and further evaluation of the impact of different surgical approaches are needed to optimize long-term outcomes. Although the different surgical techniques could be one of the potent causes, due to lack of control on this factor as an independent one in comparisons, we could not directly relate improvements in QoL scores to the modifications in the surgical technique. Interestingly, regular dental follow-ups, good oral hygiene, speech and hearing assessments, and social support might have affected the QoL scores. Our findings align with studies like Khancheza et al. and Austin, which reported that team-based management improves access to critical services, including otolaryngology care, dental services, and psychological support, all of which contribute to better QoL outcomes [31, 32]. The comparison of the impact on the other child domain was not statistically significant between the two mentioned groups. Previous studies that compared CLP children with those who had no cleft demonstrated significantly higher scores for all subscales of the impact on the child domain [25]. Khoun's study compared OHRQoL in children aged 2–8 and discovered that children with CLP had the highest mean scores in the emotional and social well-being domains [33]. Similar to our study, Pasini et al. reported that children with CLP achieved lower scores of OHIP-14 than the control group [34]. Studies showed no difference between self- and parent-reported QoL questionnaires; therefore, it was concluded that even young children had the adequacy of reporting their QoL [5, 35, 36]. Another study represented caregivers' lower scores in OHRQoL (functional well-being domain) compared to their children. Also, this discordance between the child and parents was affected by cleft type and child age [37]. Since the affected ones in the present study were very young, we decided to ask the caregivers to report OHRQoL.

In our study, the non-team patient group had higher scores than the control and team-managed groups in the total F-ECOHIS and all the subscales. This implies the importance of awareness of the cleft team members about the difficulties associated with non-team patients in their managements, and the proper guidance and supervisions for these children and their caregivers are crucial. Consistent with our study, Additionally, Veen et al. found that non-team-managed patients experience lower quality of life in domains such as family acceptance and social interactions. The present study reinforces the importance of comprehensive care models for children with CLP [38]. Also, Larson et al. reported that the internationally non-team patient group children with CLP had lower scores in speech ability than their counterparts without CLP [39].

Based on the scores in the present study, there were no significant differences between the boys and girls, consistent with previous studies that assessed the impact of sex on OHRQoL of children with CLP in the same age category [40, 41]. Bos et al. studied children aged 8–15 using the COHIP and found statistically higher scores for girls in all domains, especially in the emotional well-being and peer interaction subscales [42]. However, it should be considered that the age range was higher compared to our study. A study by Eslami et al. also demonstrated the same results [43], indicating that in the process of development and maturation, sex differences might be revealed when determining the influence of cleft on the quality of life of children. Our study was cross-sectional, limiting the ability to draw conclusions about longKal@kodi2121$$-term outcomes, particularly as esthetics and function become more prominent concerns during adolescence.

Future studies should address the long-term outcomes of multidisciplinary team management, especially during critical developmental stages. Investigating factors such as the prevalence of dental caries, access to care, and socioeconomic status, as well as the impact of surgical techniques, would further enrich understanding of the challenges faced by CLP patients.

## 5. Conclusion

In conclusion, this study highlights the positive impact of team-based management on the OHRQoL in children with UCLP. Children managed by a specialized cleft team demonstrated significantly better OHRQoL outcomes compared to those who underwent conventional surgeries, as reflected by the lower ECOHIS scores reported by caregivers. The absence of significant differences between the team-managed group and the control group further reinforces the benefits of comprehensive team management in achieving favorable outcomes. These findings underscore the importance of multidisciplinary team approaches in the surgical management of CLP to optimize patient quality of life and encourage further research into refining and expanding such care models. However, since randomization and a controlled surgical method were not possible, the improvements in quality-of-life scores cannot be directly related to the surgical method.

## Figures and Tables

**Table 1:** The median scores of cleft lip and palate patients according to their surgical technique.

F-ECOHIS	Group						*p*-Value
	Non-team patient [28]			Team-managed group [29]			
Domains	Median	Percentiles		Median	Percentiles		
		25	75		25	75	
Subscale impact on the child	39	35	40	3	1	7	<0.001
Limitation	17	15	20	0	0	0	<0.001
Psychological	7	6	9	0	0	1	<0.001
Self-image	10	6	10	0	0	0	<0.001
Symptom	5	4	5	1	0	2	<0.001
Subscale impact on the family	17	16	19	4	0	9	<0.001
Parental distress	5	4	5	1	0	2	<0.001
Family function	9	8	9	1	0	3	<0.001
Total F-ECOHIS	55	52	65	10	3	14	<0.001

Abbreviation: F-ECOHIS, Farsi version of the Early Childhood Oral Health Impact Scale.

**Table 2:** Median scores in patients without cleft and non-team patient group.

F-ECOHIS	Group						*p*-Value
	Non-team patient [28]			Control [29]			
Domains	Median	Percentiles		Median	Percentiles		
		25	75		25	75	
Subscale impact on the child	39	35	40	8	4	15	<0.001
Limitation	5	4	5	2	1	3	<0.001
Psychological	17	15	20	3	1	6	<0.001
Self-image	7	6	9	2	1	5	<0.001
Symptom	10	6	10	0	0	1	<0.001
Subscale impact on the family	9	8	9	3	2	5	<0.001
Parental distress	8	8	10	3	2	6	<0.001
Family function	17	16	19	8	4	10	<0.001
Total F-ECOHIS	55	52	65	17.03	11	24	<0.001

Abbreviation: F-ECOHIS, Farsi version of the Early Childhood Oral Health Impact Scale.

**Table 3:** Median scores in patients without cleft and the team-managed group.

F-ECOHIS	Group						*p*-Value
	Team-managed group [29]			Control [29]			
Domains	Median	Percentiles		Median	Percentiles		
		25	75		25	75	
Subscale impact on the child	3	1	7	8	4	15	0.377
Limitation	1	0	2	2	1	3	0.001
Psychological	0	0	0	3	1	6	0.017
Self-image	0	0	1	2	1	5	1.00
Symptom	0	0	0	0	0	1	0.341
Subscale impact on the family	4	0	9	8	4	10	0.505
Parental distress	0	0	3	3	2	6	0.061
Family function	1	0	3	3	2	5	0.058
Total F-ECOHIS	10	3	14	17.03	11	24	0.122

Abbreviation: F-ECOHIS, Farsi version of the Early Childhood Oral Health Impact Scale.

## Data Availability

This study's dataset is available based on a reasonable request from the corresponding author.

## Nomenclature

OHRQoL: Oral Health-Related Quality of Life
CLP: Cleft Lip and Palate
UCLP: Unilateral Cleft Lip and Palate
F-ECOHIS: Farsi Version of the Early Childhood Oral Health Impact Scale

## Supporting Information

Supporting Information The readers can see additional information regarding the comparison between girls and boys across all domains and subscales in Table S4.

## References

[1] Kappen I. F., Bittermann G. K., Stock N. M., Mink van der Molen A. B., Breugem C. C., Swanenburg de Veye H. F. (2019). Quality of Life and Patient Satisfaction in Adults Treated for a Cleft Lip and Palate: A Qualitative Analysis. *The Cleft Palate-Craniofacial Journal*.

[2] Mossey P. A., Little J., Munger R. G., Dixon M. J., Shaw W. C. (2009). Cleft Lip and Palate. *The Lancet*.

[3] Nolte F. M., Bos A., Prahl C. (2019). Quality of Life among Dutch Children with a Cleft Lip and/or Cleft Palate: A Follow-up Study. *The Cleft Palate-Craniofacial Journal*.

[4] Nidey N., Moreno Uribe L. M., Marazita M. M., Wehby G. L. (2016). Psychosocial Well-Being of Parents of Children with Oral Clefts. *Child: Care, Health and Development*.

[5] Broder H. L., Wilson-Genderson M., Sischo L. (2017). Oral Health-Related Quality of Life in Youth Receiving Cleft-Related Surgery: Self-Report and Proxy Ratings. *Quality of Life Research*.

[6] Patrick D. L., Topolski T. D., Edwards T. C. (2007). Measuring the Quality of Life of Youth With Facial Differences. *The Cleft Palate-Craniofacial Journal*.

[7] Nam Y.-T., Yun J.-W., Jun E.-J., Kim S.-S., Kim J.-B., Jeong S.-H. (2019). Evaluation of Oral Health-Related Quality of Life in Orthodontic Patients Using the Modified Oral Impact on Daily Performance (OIDP) Questionnaire. *Journal of Korean Academy of Oral Health*.

[8] Antunes L. S., Maués C. P. R., Nadaes M. R., Costa M. C., Küchler E. C., Antunes L. A. A. (2014). The Impact of Nonsyndromic Oral Clefts on Family Quality of Life. *Special Care in Dentistry*.

[9] Bruneel L., Bettens K., Van Lierde K. (2019). The Relationship between Health-Related Quality of Life and Speech in Patients with Cleft Palate. *International Journal of Pediatric Otorhinolaryngology*.

[10] Kramer F.-J., Gruber R., Fialka F., Sinikovic B., Schliephake H. (2008). Quality of Life and Family Functioning in Children with Nonsyndromic Orofacial Clefts at Preschool Ages. *Journal of Craniofacial Surgery*.

[11] Téblick S., Ruymaekers M., Van de Casteele E., Nadjmi N. (2019). Effect of Cleft Palate Closure Technique on Speech and Middle Ear Outcome: A Systematic Review. *Journal of Oral and Maxillofacial Surgery*.

[12] Sinko K., Cede J., Jagsch R. (2017). Facial Aesthetics in Young Adults after Cleft Lip and Palate Treatment over Five Decades. *Scientific Reports*.

[13] Emeka C. I., Adeyemo W. L., Ladeinde A. L., Butali A. (2017). A Comparative Study of Quality of Life of Families with Children Born with Cleft Lip and/or Palate before and after Surgical Treatment. *Journal of the Korean Association of Oral and Maxillofacial Surgeons*.

[14] Kiyak H. A. (2008). Does Orthodontic Treatment Affect Patients’ Quality of Life?. *Journal of Dental Education*.

[15] Pahel B. T., Rozier R. G., Slade G. D. (2007). Parental Perceptions of Children’s Oral Health: The Early Childhood Oral Health Impact Scale (ECOHIS). *Health and Quality of Life Outcomes*.

[16] Tuominen M. L., Tuominen R. J. (2009). Factors Associated with Subjective Need for Orthodontic Treatment Among Finnish University Applicants. *Acta Odontologica Scandinavica*.

[17] Åstrøm A., Haugejorden O., Skaret E., Trovik T., Klock K. (2005). Oral Impacts on Daily Performance in Norwegian Adults: Validity, Reliability and Prevalence Estimates. *European Journal of Oral Sciences*.

[18] Srisilapanan P., Sheiham A. (2001). The Prevalence of Dental Impacts on Daily Performances in Older People in Northern Thailand. *Gerodontology*.

[19] Purohit B. M., Singh A., Acharya S., Bhat M., Priya H. (2012). Assessment and Validation of the Oral Impact on Daily Performance (OIDP) Instrument Among Adults in Karnataka, South India. *Community Dental Health*.

[20] Zhang M., McGrath C., Hägg U. (2007). Patients’ Expectations and Experiences of Fixed Orthodontic Appliance Therapy: Impact on Quality of Life. *The Angle Orthodontist*.

[21] Sischo L., Wilson-Genderson M., Broder H. L. (2017). Quality-of-Life in Children with Orofacial Clefts and Caregiver Well-Being. *Journal of Dental Research*.

[22] Campbell S. B. (1995). Behavior Problems in Preschool Children: A Review of Recent Research. *Journal of Child Psychology and Psychiatry*.

[23] Zeraatkar M., Ajami S., Nadjmi N., Golkari A. (2018). Impact of Oral Clefts on the Oral Health-Related Quality of Life of Preschool Children and Their Parents. *Nigerian Journal of Clinical Practice*.

[24] Saif S., Serhier Z., Bourzgui F. (2019). The Quality of Life of Children with and Without Lip and Palate Clefts Before and After Dental Treatment Using the Moroccan Version of ECOHIS. *International Journal of Drug Research and Dental Science*.

[25] Rando G. M., Jorge P. K., Vitor L. L. R. (2018). Oral Health-Related Quality of Life of Children with Oral Clefts and Their Families. *Journal of Applied Oral Science*.

[26] Nadjmi N. (2018). *Surgical Management of Cleft Lip and Palate: A Comprehensive Atlas*.

[27] Jabarifar S.-E., Golkari A., IJadi M. H., Jafarzadeh M., Khadem P. (2010). Validation of a Farsi Version of the Early Childhood Oral Health Impact Scale (F-ECOHIS). *BMC Oral Health*.

[28] Filgueira I. G., Azevedo I. D., da Silva Ramalho L., Gomes P. N., Rêgo D. M. (2015). Quality of Life of Patients with Cleft Lip and/or Cleft Palate: Perspective of Parents/Guardians. *Pesquisa Brasileira Em Odontopediatria e Clínica Integrada*.

[29] Webb A. A., Watts R., Read-Ward E., Hodgkins J., Markus A. F. (2001). Audit of a Multidisciplinary Approach to the Care of Children with Unilateral and Bilateral Cleft Lip and Palate. *British Journal of Oral and Maxillofacial Surgery*.

[30] Defabianis P., Ninivaggi R., Romano F. (2022). Influence of Cleft Lip and Palate on Oral Health-Related Quality of Life in Northern Italy: Exploring Both the Children’s and Caregivers’ Perspectives. *Children (Basel, Switzerland)*.

[31] Khanchezar F., Moradi N., Tahmasebi Fard N., Latifi S. M., Bassak Nejad S., Hosseini Beidokhti M. (2019). The Effect of Teamwork on Children With Cleft Lip and Palate and Their Mother’s Quality of Life. *The Cleft Palate-Craniofacial Journal*.

[32] Austin A. A., Druschel C. M., Tyler M. C., Romitti P. A., West Damiano, etal P. C. (2010). Interdisciplinary Craniofacial Teams Compared with Individual Providers: Is Orofacial Cleft Care More Comprehensive and Do Parents Perceive Better Outcomes?. *The Cleft Palate-Craniofacial Journal*.

[33] Khoun T., Malden P. E., Turton B. J. (2018). Oral Health-Related Quality of Life in Young Cambodian Children: A Validation Study with a Focus on Children With Cleft Lip and/or Palate. *International Journal of Paediatric Dentistry*.

[34] Pasini M., Cagidiaco I., Fambrini E., Miceli M., Carli E. (2022). Life Quality of Children Affected by Cleft Lip Palate and Alveolus (CLPA). *Children (Basel, Switzerland)*.

[35] Yusof M. S., Mohd Ibrahim H. (2023). The Impact of Cleft Lip and Palate on the Quality of Life of Young Children: A Scoping Review. *The Medical Journal of Malaysia*.

[36] Konan P., Manosudprasit M., Pisek P., Pisek A., Wangsrimongkol T. (2015). Oral Health-Related Quality of Life in Children and Young Adolescent Orthodontic Cleft Patients. *Journal of the Medical Association of Thailand = Chotmaihet Thangphaet*.

[37] Defabianis P., Cogo C., Massa S., Romano F. (2022). Oral-Health-Related Quality of Life among Non-Syndromic School-Age Children with Orofacial Clefts: Results from a Cross-Sectional Study in Northern Italy. *Children (Basel, Switzerland)*.

[38] van Veen M. M., van den Berge B. A., Mouës-Vink C. M. (2022). Quality of Life of Adopted Chinese Versus Nonadopted Dutch Children with Cleft Lip and/or Palate: A Propensity Score Matched Analysis. *The Cleft Palate Craniofacial Journal*.

[39] Larsson A. K., Persson C., Klintö K., Miniscalco C. (2021). Internationally Adopted Children with and without a Cleft Lip and Palate Showed No Differences in Language Ability at School-Age. *Acta Paediatrica*.

[40] Awoyale T., Onajole A. T., Ogunnowo B. E., Adeyemo W. L., Wanyonyi K. L., Butali A. (2016). Quality of Life of Family Caregivers of Children With Orofacial Clefts in Nigeria: A Mixed-Method Study. *Oral Diseases*.

[41] Ajami S., Toraby F., Shavakhi M., Eslami N. (2018). The Impact of Type-d Personality on Oral Health-Related Quality of Life in Cleft Lip and Palate Adolescents. *Journal of Craniofacial Surgery*.

[42] Bos A., Hoogstraten J., Zentner A. (2010). Perceptions of Dutch Orthodontic Patients and Their Parents on Oral Health-Related Quality of Life. *The Angle Orthodontist*.

[43] Eslami N., Majidi M. R., Aliakbarian M., Hasanzadeh N. (2013). Oral Health-Related Quality of Life in Children With Cleft Lip and Palate. *Journal of Craniofacial Surgery*.

