# Supplementary material for: Impact of Multidisciplinary Cleft Team Care on Oral Health Quality of Life in Children With Unilateral Cleft Lip and Palate: A Focus on Early Intervention vs. Sporadic Treatment
Source: Int J Dent. 2025 Mar 19;2025:1642111. doi: 10.1155/ijod/1642111 (PMC11944874; doi:10.1155/ijod/1642111)
Supplement: Supporting Information — The readers can see additional information regarding the comparison between girls and boys across all domains and subscales in Table S4. [file 1642111.f1.docx]

**Table 4. Median scores according to the sex**

| **F-ECOHIS**  **Domains** | **Group** | | | | | | | | | | | | P-Value |
| --- | --- | --- | --- | --- | --- | --- | --- | --- | --- | --- | --- | --- | --- |
|  | **Non-team patient** | | | | **Team-managed** | | | | **Control** | | | |  |
|  | **Sex** | | | | **Sex** | | | | **Sex** | | | |  |
|  | **Male** | | **Female** | | **Male** | | **Female** | | **Male** | | **Female** | |  |
|  | **Median** | **Q_1_-Q_3_** | **Median** | **Q_1_-Q_3_** | **Median** | **Q_1_-Q_3_** | **Median** | **Q_1_-Q_3_** | **Median** | **Q_1_-Q_3_** | **Median** | **Q_1_-Q_3_** |  |
| **Subscale impact on the child** | 37 | 35–39 | 39 | 39–40 | 4 | 2–8 | 3 | 1–6 | 8 | 5–15 | 9 | 3–13 | 0.401 |
| Limitation | 5 | 4–5 | 5 | 5–5 | 1 | 0–2 | 1 | 0–2 | 2 | 1–3 | 2 | 1–3 | 0.939 |
| Psychological | 17 | 15–19 | 17 | 17–20 | 0 | 0–0 | 0 | 0–0 | 4 | 2–6 | 3 | 1–6 | 0.588 |
| Self-image | 7 | 6–9 | 7 | 7–9 | 0 | 0–1 | 0 | 0–1 | 2 | 1–6 | 2 | 0–3 | 0.463 |
| Symptom | 10 | 6–10 | 10 | 6–10 | 1 | 0–0 | 0 | 0–0 | 0 | 0–1 | 0 | 0–2 | 0.824 |
| **Subscale impact on the family** | 17 | 16–19 | 17 | 16–20 | 4 | 3–9 | 0 | 0–9 | 8 | 6–11 | 6 | 3–10 | 0.732 |
| Parental distress | 8 | 8–10 | 8 | 8–10 | 1 | 0–3 | 0 | 0–2 | 4 | 3–6 | 3 | 2–6 | 0.447 |
| Family function | 9 | 8–9 | 9 | 8–10 | 1 | 0–3 | 1 | 0–2 | 5 | 3–6 | 3 | 1–4 | 0.440 |
| Total F-ECOHIS | 55 | 52–56 | 55 | 55–60 | 11 | 3–14 | 9 | 3–16 | 16 | 11–24 | 15 | 6–22 | 0.653 |

F-ECOHIS=Farsi version of the Early Childhood Oral Health Impact Scale
